# *Strongyloides stercoralis* Infection in Humans in West Africa, 1975–2024: Systematic Review and Meta-Analysis

**DOI:** 10.3390/tropicalmed10110321

**Published:** 2025-11-17

**Authors:** Rufin K. Assaré, Mamadou Ouattara, Sören L. Becker, Fidèle K. Bassa, Nana R. Diakité, Jürg Utzinger, Eliézer K. N’Goran

**Affiliations:** 1 Unité de Formation et de Recherche Biosciences, Université Félix Houphouët-Boigny, Abidjan 22 BP 582, Côte d’Ivoire; mamadou_ouatt@yahoo.fr (M.O.); fidelebassa@ymail.com (F.K.B.); diaknarose@yahoo.fr (N.R.D.); eliezerngoran@yahoo.fr (E.K.N.); 2 Environnement et Santé, Centre Suisse de Recherches Scientifiques en Côte d’Ivoire, Abidjan 01 BP 1303, Côte d’Ivoire; 3 Epidemioloy and Public Health, Swiss Tropical and Public Health Institute, Kreuzstrasse 2, CH-4123 Allschwil, Switzerland; soeren.becker@uks.eu (S.L.B.); juerg.utzinger@swisstph.ch (J.U.); 4 Faculty of Medicine, University of Basel, CH-4003 Basel, Switzerland; 5 Institute of Medical Microbiology and Hygiene, Saarland University, Kirrberger Strasse, Building 43, 66421 Homburg, Germany

**Keywords:** diagnosis, meta-analysis, prevalence, *Strongyloides stercoralis*, strongyloidiasis, systematic review, West Africa

## Abstract

Strongyloidiasis is an underappreciated helminth infection that belongs to a group of neglected tropical diseases. The aim of this systematic review and meta-analysis was to determine the pooled prevalence of *Strongyloides stercoralis* infection in humans in 16 West African countries. We searched African Journals Online, Embase, Horizon, Google Scholar, ProQuest, PubMed, Scopus, and Web of Science to identify articles assessing *S. stercoralis* prevalence data. The search was restricted to articles published between 1 January 1975 and 31 December 2024 without language restriction. We followed the PRISMA guidelines. A total of 21,250 articles were identified, 336 of which met the inclusion criteria. The most frequently used diagnostic tools were Kato-Katz (35.1%) and formol-ether coprological methods (23.4%). Strongyloidiasis was reported in 15 of the 16 West African countries; Mali was the only country where it was absent. The *S. stercoralis* regional prevalence was 4.4%, ranging from 0.2% in Burkina Faso to 18.9% in The Gambia. *S. stercoralis* infection prevalence decreased from 14.0% (1975–1984) to 4.1% (2015–2024). *S. stercoralis* prevalence showed strong heterogeneity with the highest prevalence mainly observed in countries in the Gulf of Guinea. Most of the employed diagnostic techniques were inappropriate; the reported *S. stercoralis* prevalence is, thus, likely an underestimation of the true situation. Our observations call for more sensitive *S. stercoralis* diagnostic tools and strategies for strongyloidiasis control that are tailored to the different social-ecological settings of West Africa.

## 1. Introduction

Strongyloidiasis is a neglected parasitic worm infection, which is predominantly caused by the nematode species *Strongyloides stercoralis* [1]. A recent review using spatio-temporal statistical modelling estimated that more than 600 million people might be infected with *S. stercoralis* worldwide [2]. Strongyloidiasis shows the highest prevalence in tropical and subtropical countries, particularly in areas with poor sanitation and hygiene conditions. Most often, *S. stercoralis* infection causes no overt symptomatology. However, chronic infection might cause diarrhoea, abdominal pain, skin rashes, Loeffler’s syndrome, dry cough, recurrent sore throat, loss of appetite, blood in stool, anaemia, heartburn, and bloating. In immunosuppressed people, strongyloidiasis can lead to hyperinfection syndromes and dissemination, which can be fatal. Infection with human T-lymphotropic virus 1 (HTLV-1), hypogammaglobulinaemia, malnutrition, corticosteroid consumption, diabetes mellitus, alcoholism, and chronic renal failure increase the risk of severe *S. stercoralis* infection [3,4,5].

Humans are primarily infected by *S. stercoralis* through the cutaneous route. Filariform larvae penetrate the skin of the host when walking barefoot. The larvae migrate to the lung, before finally reaching the intestine where the parthenogenic parasitic female worm produces eggs. The eggs rapidly hatch into rhabditiform larvae inside the host’s gut. Some rhabditiform larvae are excreted in soil and give rise to infectious filariform larvae, which are able to penetrate skin and restart the life cycle of the worm. The remaining rhabditiform larvae develop into filariform larvae in the host’s gut and penetrate the bowel wall, thereby establishing the autoinfective lifecycle. The helminth’s almost unique characteristic of parthenogenesis and autoinfection provides the basis for lifelong persistent infection, which can lead to hyperinfection.

Although ivermectin is recommended as an efficacious drug, control programmes for strongyloidiasis are lacking in endemic countries, particularly in sub-Saharan Africa. An important reason is that the prevalence of *S. stercoralis* infection is unknown or underestimated. Strongyloidiasis diagnosis is based on the identification of larvae in stool samples. However, most of the widely used diagnostic tools have low sensitivity because no eggs, only larvae, are shed in the faeces. More sensitive diagnostic approaches such as Koga agar plate (KAP) culture, Baermann, and polymerase chain reaction (PCR), are rarely used in field surveys in resource-constrained settings [6,7,8]. The Kato-Katz thick smear technique (KK) and direct faecal smear analysis have been mainly used in epidemiological surveys, although these techniques are better suited to detect helminth eggs than larvae; hence, they result in low *S. stercoralis* prevalence estimates. Over the past decade, several studies were conducted to assess the *S. stercoralis* prevalence. The prevalence of infection in the general population was estimated, and key risk factors were determined [2,3,9]. A recent review aimed to deepen the understanding of the epidemiology of strongyloidiasis in Africa based on different diagnostic techniques, study settings, and populations studied [10]. Considering cross-sectional surveys, the review reported data of *S. stercoralis* for five West African countries. In two of the included countries (i.e., Burkina Faso and Senegal), only one survey met the inclusion criteria. However, historical data on strongyloidiasis are available that date back to the 1970s for many West African countries.

The purpose of the current systematic review and meta-analysis was to determine the prevalence of *S. stercoralis* infection in West Africa stratified by country, decade, diagnostic method, sample size, study setting, and population. The results are important and might guide strongyloidiasis-control efforts in West Africa and elsewhere.

## 2. Materials and Methods

### 2.1. Search Strategy

The systematic review was conducted following the Preferred Reporting Items for Systematic Reviews and Meta-Analyses (PRISMA) guidelines (Table S1). The review protocol is available on the International Prospective Register of Systematic Reviews (PROSPERO) under registration number CRD42022332324.

We searched for relevant publications pertaining to *S. stercoralis* infection in West African countries, using the following databases: African Journals Online, Embase, Horizon, Google Scholar, ProQuest, PubMed, Scopus, and Web of Science. The search was restricted to studies on humans published between 1 January 1975 and 31 December 2024. There was no language restriction. The keywords “strongyloidiasis” and “parasitose” were used to search African Journals Online and Horizon, respectively. In Google Scholar, the search terms were “anguillulose” and “Strongyloides”, combined with the countries of West Africa (i.e., Benin, Burkina Faso, Cape Verde, Côte d’Ivoire, The Gambia, Ghana, Guinea, Guinea-Bissau, Liberia, Mali, Mauritania, Niger, Nigeria, Senegal, Sierra Leone, and Togo). For the remaining five online databases (i.e., Embase, ProQuest, PubMed, Scopus, and Web of Science), the search terms included the following Medical Subject Headings (MeSH): “strongyloidiasis”, “anguilllulose”, “anguillulosis”, “*S. stercoralis*”, “soil-transmitted helminth”, “anguillul*”, “strongyl*”, and “helminth*”. These search terms were used in combination with the Boolean operators “OR”, “AND”, and the name of the West African countries.

### 2.2. Selection Criteria

Studies were selected for the current review if: (i) they were observational or clinical studies; (ii) they were carried out in West African countries; (iii) had human stool or blood samples examined; and (iv) studies reported prevalence of *S. stercoralis* infection or provided data on sample size and number of infected individuals. We excluded studies that focused on animal infections, reviews, commentaries, editorials (expert opinions), and symposia. Studies conducted on people coming from West Africa but living outside this region were also excluded. Additionally, articles were excluded if the full text was not available.

### 2.3. Data Extraction

The first author conducted the literature search. A second researcher double-checked the recorded articles based on the study inclusion and exclusion criteria. In case of discrepancies, a third researcher was consulted until agreement was reached.

The following information was retrieved: first author, year of publication, location, country, study design, study setting, study population, time of field work, diagnostic method, number of male participants, number of *S. stercoralis*-positive males, number of female participants, number of *S. stercoralis*-positive females, individual sample size, and number of *S. stercoralis*-positive individuals, *S. stercoralis* prevalence, and 95% confidence intervals (CIs) were estimated.

### 2.4. Quality Assessment

The quality of the included studies was assessed, using the checklist of Joanna Briggs Institute (JBI) for quality assessment of prevalence studies tools [11]. This checklist is a questionnaire-based method with nine specific items; namely (i) sample frame appropriate to address the target population; (ii) study participants sampled in an appropriate way; (iii) adequate sample size; (iv) study subjects and the setting described in detail; (v) data analysis conducted with sufficient coverage of the identified sample; (vi) valid methods used for the identification of the condition; (vii) the conditions measured in a standard and reliable way for all participants; (viii) appropriate statistical analysis; and (ix) adequate response rate. The responses were scored as 1 indicating “yes” and 0 meaning “not reported”. For each included article, the total score of “yes” responses were calculated and classified into three categories, as follow: ‘high risk of bias’ (low quality), ‘moderate risk of bias’ (moderate quality), and ‘low risk of bias’ (high quality) if the total scores of “yes” was <5, 5–7, and 8–9, respectively. Low quality articles were included in the final analysis.

### 2.5. Statistical Analysis

Data entry was performed using an Excel 2016 spreadsheet, while the meta-analysis was performed using STATA version BE 18.0 (Stata Corporation; College Station, TX, USA). The prevalence of *S. stercoralis* infection and its 95% CI were calculated using the exact binomial interval (http://statpages.info/confint.html. Accessed from 20 January 2025 to 15 February 2025). Cochran’s Q test and *I*^2^ statistic test were utilised to assess for heterogeneity among the included studies. The heterogeneity was considered as low, moderate, or high when the *I*^2^ value was above 25%, 50%, and 75%, respectively. Cochran’s Q test with *p*-value < 0.10 confirmed that there was statistically significant heterogeneity between the studies. Forest plots were generated to estimate the pooled effect size and to display a visual summary of the data. The correlation between strongyloidiasis and risk factors was measured based on the odds ratio and corresponding 95% CI. Funnel plots were generated, and Egger’s regression test was performed to assess publication bias between studies. *p*-value < 0.05 corresponds to the presence of publication bias across studies.

Subgroup analysis was conducted by decade of publication, country, diagnostic method, study population, categories of sample size, and study setting. A map of strongyloidiasis prevalence in West African countries was generated using QGIS version 3.4 Madeira (QGIS Development Team, Open Source Geospatial Foundation).

## 3. Results

### 3.1. Study Selection

**Figure 1:**
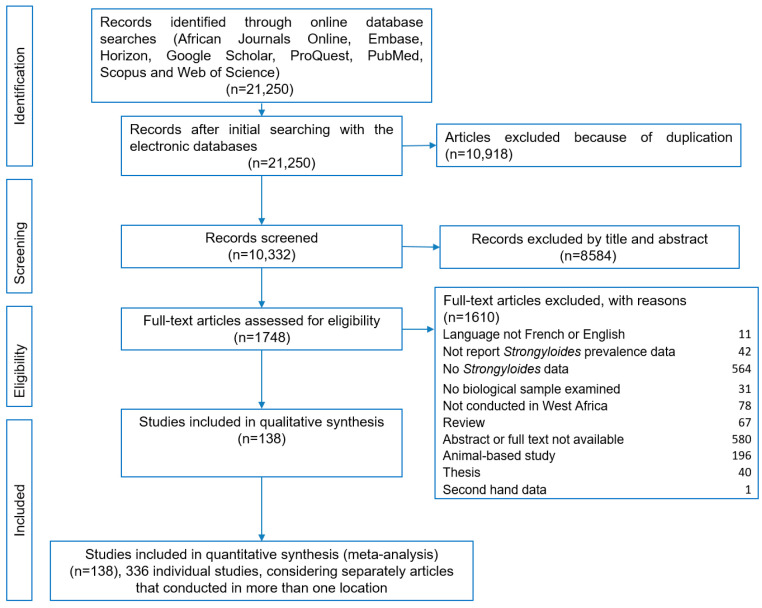
Flow diagram of studies pertaining to *Strongyloides stercoralis* infection in West Africa from 1975 to 2024 for the systematic review and meta-analysis.

A total of 21,250 articles were identified through our search of the eight electronic databases (Figure 1). After removing 10,918 duplicates, another 8584 articles were excluded based on careful study of the abstract. Full text analysis of the remaining articles resulted in the exclusion of another 1609 articles due to different reasons. Overall, 138 articles fulfilled our inclusion criteria and 11 (8.0%) of these articles were specifically designed to assess *S. stercoralis* infection. Considering, separately, articles that were conducted in more than one setting, 336 individual studies were included in this systematic review and meta-analysis.

### 3.2. Characteristics of the Included Studies

**Figure 2:**
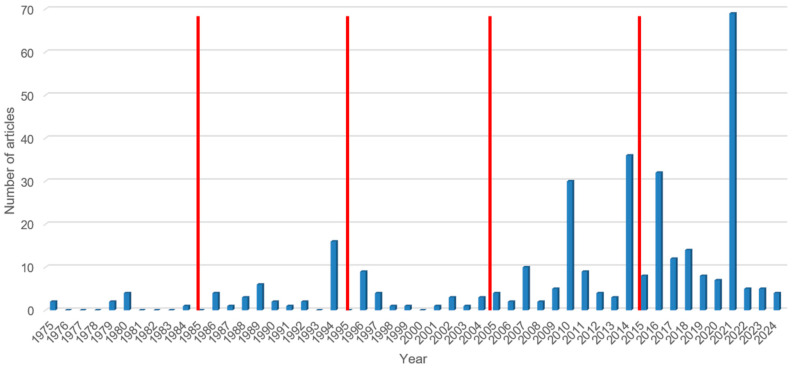
Number of publications pertaining to *Strongyloides stercoralis* infection in West Africa from 1975 to 2024. The red lines indicate the decadal limits.

The detailed characteristics of the included studies are summarised in Table 1. We found data for 15 of the 16 West African countries. In Mali, no data for *S. stercoralis* were reported. The highest number of individual studies was reported from Nigeria (n = 119), followed by Benin (n = 67), and Côte d’Ivoire (n = 46). Only one individual study was carried out in each of the two countries Cape Verde and Niger. The highest number of individual studies was published in 2021 (n = 69), followed by 2014 (n = 36), and 2016 (n = 32) (Figure 2). A total of 143,112 individuals were enrolled in the selected studies (Table 1). More than half (193 out of 336) of the included individual studies focused on school-aged children, whereas 13 individual studies assessed strongyloidiasis specifically in females. Approximately three-fifths (196/336) of the individual studies were conducted in schools, whereas the remaining individual studies were carried out in the community (n = 98) or in hospital settings (n = 42).

**Figure 3:**
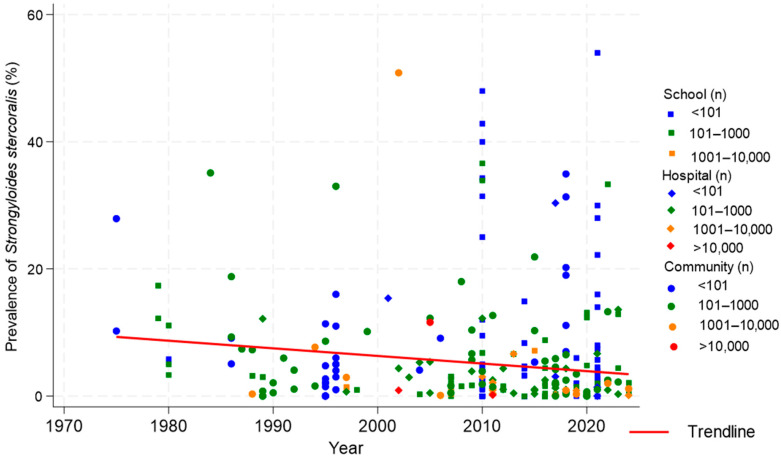
Dynamic of *Strongyloides stercoralis* infection prevalence in community-, hospital-, and school-based studies in West Africa from 1975 to 2024. n: Number of participants.

Most of the individual studies (n = 166) had sample sizes lower than or equal to 100 participants (Table 2). The highest sample size was 20,250 participants (a study conducted in the North and Upper Eastern Region of Ghana), while the lowest number of participants were enrolled in a study in Ujagba, Nigeria (9 participants) and Diowle, Mauritania (8 participants; Table S2). The prevalence of *S. stercoralis* infection of the included individual studies ranged from 0% to 54.0%. The infection prevalence of *S. stercoralis* decreased from 14.0% (95% CI: 6.9–21.2%) in the period 1975–1984 to 3.8% (95% CI: 3.2–4.3%) in 2015–2024 (Figure 3). Considering 2010 as the time limit and 20% as the *S. stercoralis* prevalence limit; the proportion of individual studies where the infection prevalence exceeded 20% after 2010 (4.6%) was more than two times lower than the corresponding value (10.0%) from 1975 to 2010 (Figure 3). Most of the highest sample sizes (>1000 participants) were reported after 2000 (80.8%, 21/26). The individual studies were predominantly carried out in schools.

**Figure 4:**
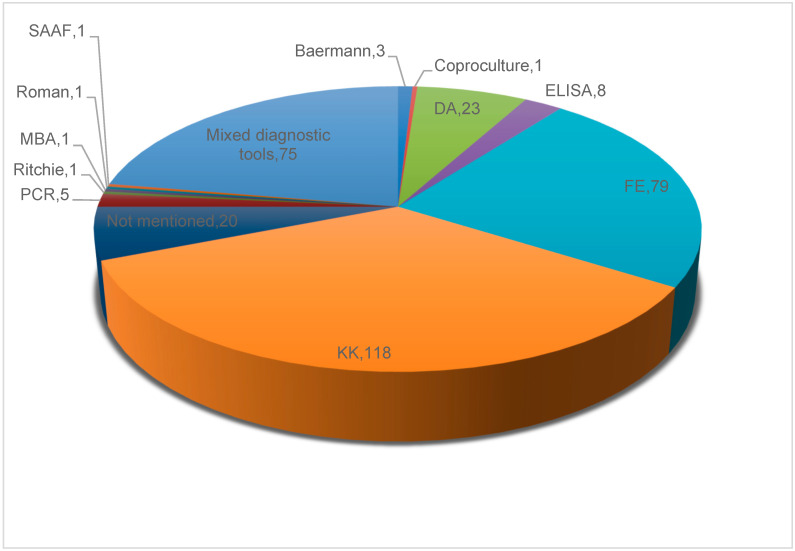
Number and percentage of individual studies using specific diagnostic tests for the detection of *Strongyloides stercoralis* infection in West Africa from 1975 to 2024. DA: direct analysis, FE: formol-ether, KK: Kato-Katz, MBA: multiplex bead assay, SAAF: sodium acetic acid formalin.

The KK technique (n = 118, 35.1%) was the predominant diagnostic approach, followed by the formol-ether (FE) method (n = 79, 23.5%) (Figure 4).

### 3.3. Quality of Studies

Table S3 summarises the results of the included articles’ quality assessment. The quality score of the articles ranged from 4 to 9 on a scale from 0 to 9. Approximately, four-fifths (n = 110) of the included articles were of moderate quality, while 19.6% (27 articles) and 0.7% (n = 1) were of high and low quality, respectively. The average score was 6.6 and shows that the overall quality of the publications included in this analysis was moderate.

### 3.4. Publication Bias

Funnel plot symmetry indicated the presence of publication bias among the included individual studies (Figure S1A). Egger’s regression test result revealed a significant publication bias across the included individual studies that were meta-analysed (*p* < 0.001).

### 3.5. Meta-Regression Analysis

The overall pooled prevalence of *S. stercoralis* infection in West Africa was 4.4% (95% CI: 4.1–4.8%). There was considerable heterogeneity across the individual studies selected in this review (*I*^2^ = 96.8%, *p* < 0.001). Our meta-regression analysis revealed that the sample size of the study population (regression coefficient: 0.42, 95% CI: 0.34–0.53, *p* < 0.001) significantly contributed to the heterogeneity across the included individual studies (Figure S1B). In contrast, decade of publication (*p* = 0.141), study country (*p* = 0.812), diagnostic method used (*p* = 0.540), study population (*p* = 0.478), and study setting (*p* = 0.433) were no significant sources of heterogeneity among the selected individual studies (Table 3).

### 3.6. Strongyloidiasis in West Africa

**Figure 5:**
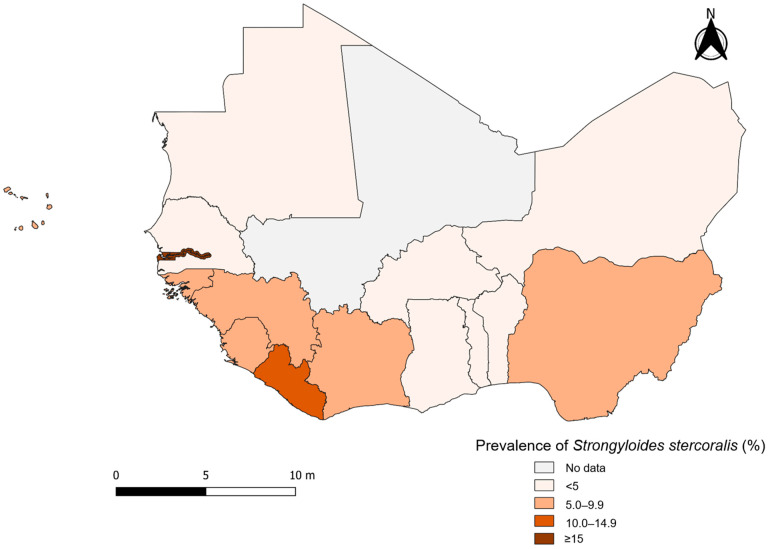
Prevalence of *Strongyloides stercoralis* infection in West African countries between 1975 and 2024.

Figure 5 displays the pooled prevalence of strongyloidiasis across West Africa. The highest pooled prevalence of *S. stercoralis* infection was reported in The Gambia (≥15%), followed by Liberia and Togo with an estimated prevalence ranging from 10.0% to 14.9%. *S. stercoralis* prevalence ranged from 5.0% to 9.9% in Cape Verde, Côte d’Ivoire, Guinea, Guinea-Bissau, Nigeria, and Sierra Leone. In the remaining six countries, the prevalence of *S. stercoralis* infection was below 5%.

**Figure 6:**
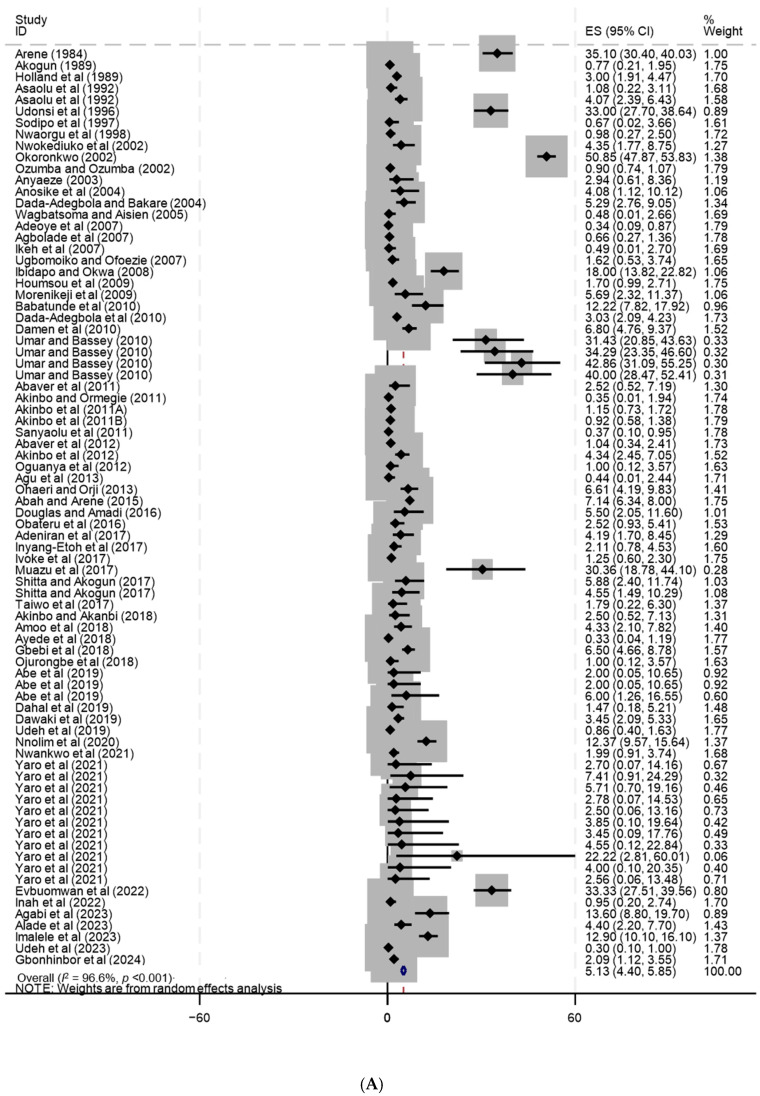

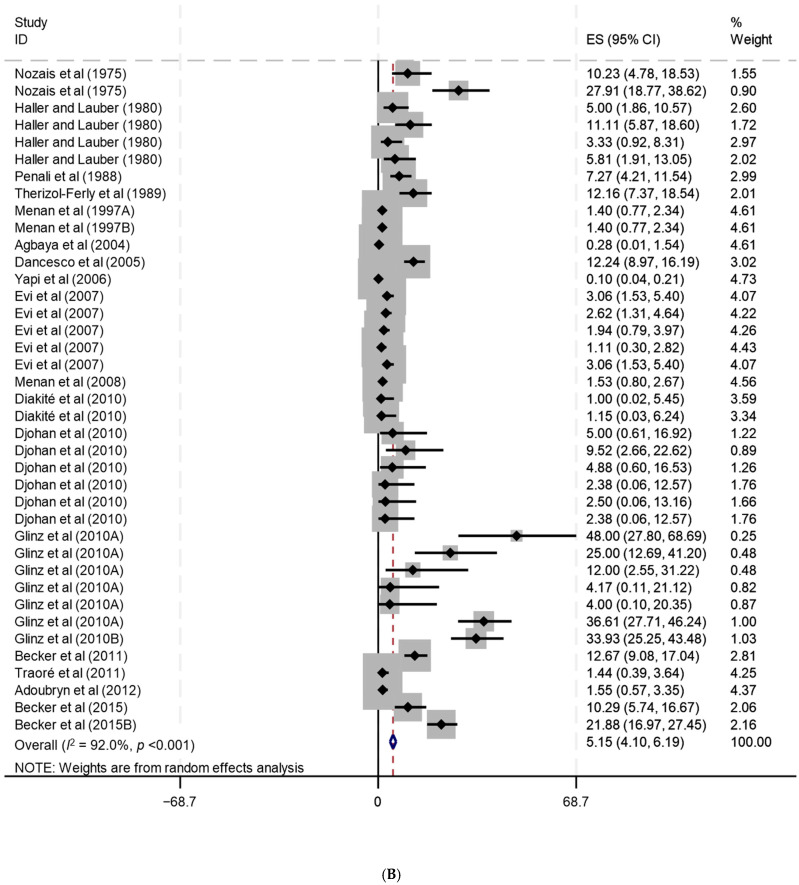

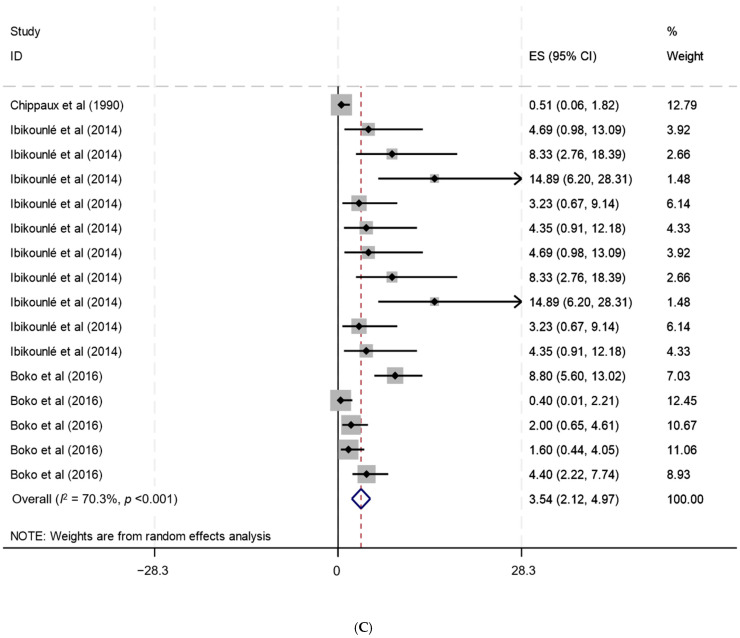

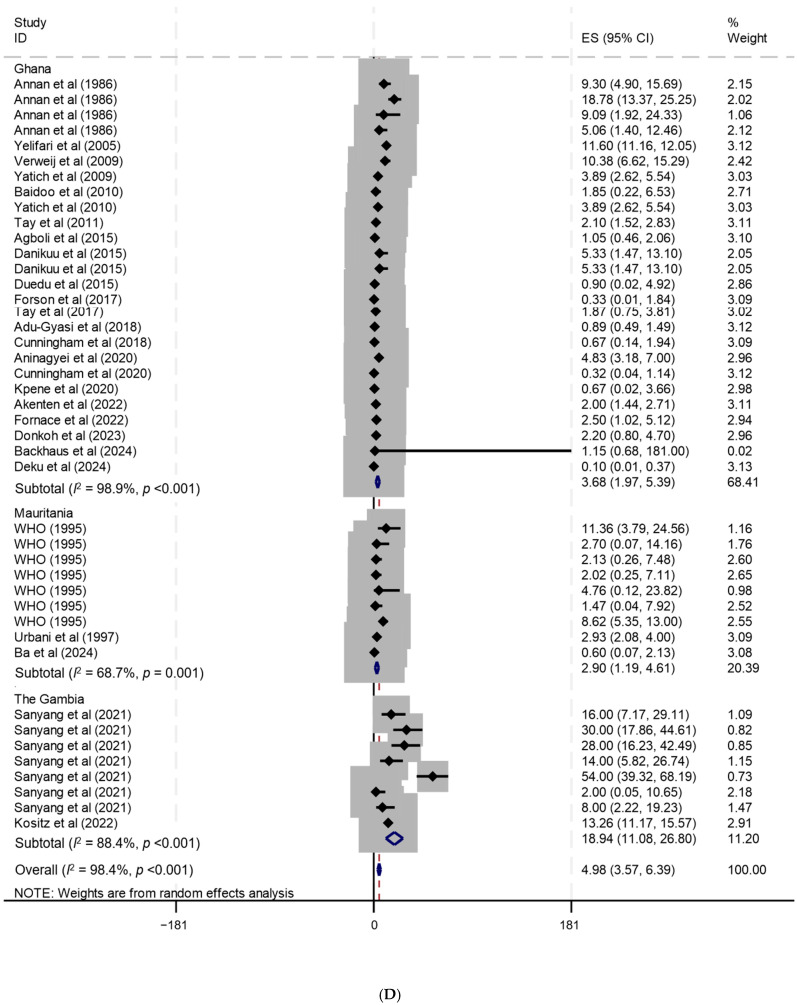

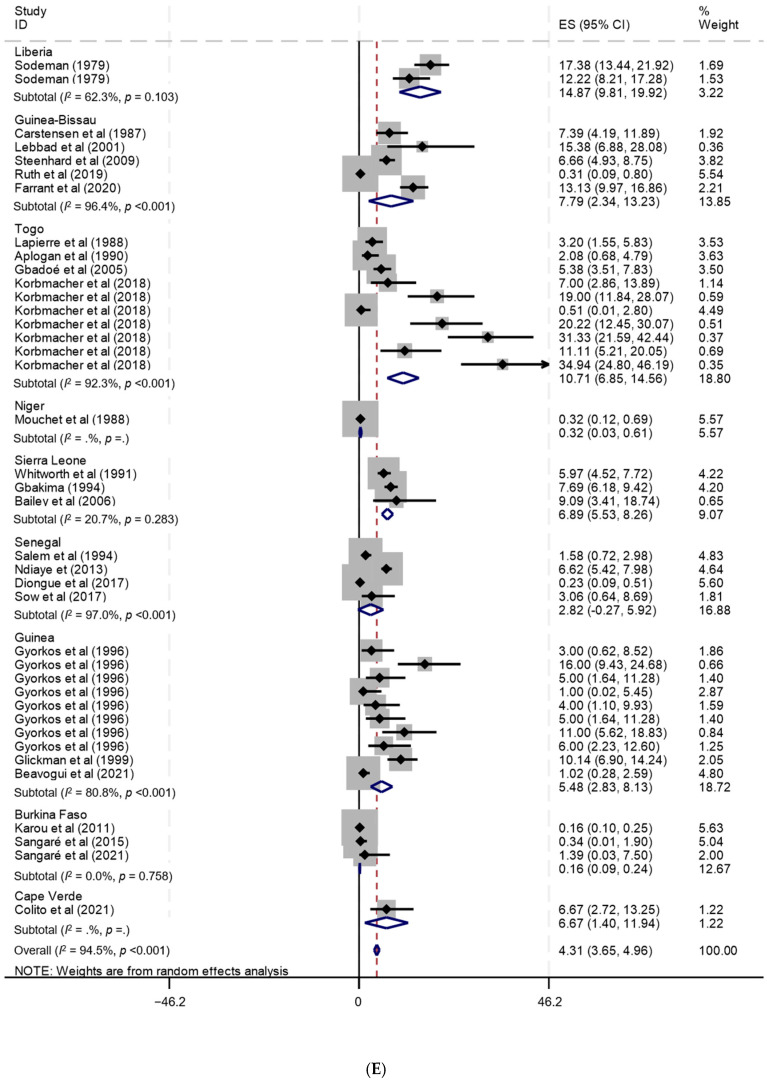
(**A**) Prevalence of *Strongyloides stercoralis* infection in Nigeria [51,52,53,54,55,56,57,58,59,60,61,62,63,64,65,66,67,68,69,70,71,72,73,74,75,76,77,78,79,80,81,82,83,84,85,86,87,88,89,90,91,92,93,94,95,96,97,98,99,100,101,102,103,104,105,106,107,108,109,110,111,112,113]. (**B**) Prevalence of *Strongyloides stercoralis* infection in Côte d’Ivoire [31,32,33,34,35,36,37,38,39,40,41,42,43,44,45,46,47,48,49,50]. (**C**) Prevalence of *Strongyloides stercoralis* infection ienin [136,137,138]. (**D**) Prevalence of *Strongyloides stercoralis* infection in Ghana, Mauritania, and The Gambia [12,13,114,115,116,117,118,119,120,121,122,123,124,125,126,127,128,129,130,131,132,133,134,135,139,140,141]. (**E**) Prevalence of *Strongyloides stercoralis* infection in Burkina Faso, Cape Verde, Guinea, Guinea-Bissau, Liberia, Niger, Senegal, Sierra Leone, and Togo [14,15,16,17,18,19,20,21,22,23,24,25,26,27,28,29,30,141,142,143,144,145,146,147,148]. Grey square represents the weight of each study in the meta-analysis. The blue diamond displays overall or subtotal pooled prevalence of *S. stercoralis* infection. The width of the diamond shows the 95% CI of the pooled prevalence of the infection.

Table 4 summarises the pooled *S. stercoralis* infection prevalence by country, while the visual presentation of these findings are shown in Figure 6A–E. The highest pooled prevalence of *S. stercoralis* infection was reported from The Gambia when using KK (18.9%, 95% CI: 11.1–26.8%, high heterogeneity: *I*^2^ = 88.4%, *p* < 0.001). The second highest prevalence was found in Liberia (14.9%, 95% CI: 9.8–19.9%, *I*^2^ = 62.3%, *p* = 0.103), and the third in Togo (10.7%, 95% CI: 6.9–92.3%, *I*^2^ = 92.3, *p* < 0.001). In Liberia, direct analysis (DA) was used as diagnostic tool, while in Togo, 7 out of 10 individual studies employed an enzyme-linked immunosorbent assay (ELISA) technique. Almost all the individual studies in The Gambia (18 out of 19 individual studies) and the individual studies in Liberia (2 studies) were conducted at schools.

The lowest pooled prevalence of *S. stercoralis* was 0.2% (95% CI: 0.1–0.2%) and 0.3% (95% CI: 0.0–0.6%) recorded in Burkina Faso and Niger, respectively. In Burkina Faso, the selected individual studies (n = 5) were carried out in hospitals, three of them targeting females. A combination of DA, Ritchie, and Willis methods were used in three individual studies, a combination of DA and FE was used in one study, and DA was used in one individual study in Burkina Faso. The study in Niger was community-based and used the FE method for diagnosis. In Nigeria, a relative higher number of participants 47,705 (33.3%) were enrolled in 119 individual studies and the pooled prevalence was 5.1% (95% CI: 4.4–5.9%) with considerable heterogeneity (*I*^2^ = 96.6%, *p* < 0.001). Approximately three-fifths of the individual studies (69/119 individual studies) were school-based and 61.0% (72/119 individual studies) used FE concentration method.

### 3.7. Subgroup Analysis

#### 3.7.1. *S. stercoralis* Prevalence by Decade of Publication

Forest plots using random effects model indicated that the highest pooled *S. stercoralis* prevalence was 14.0% (95% CI: 6.9–21.2%) reported from 1975 to 1984 (Figure S2A,B). The lowest prevalence of infection was 3.8% (95% CI: 3.2–4.3%) recorded in the years 2015–2024 (Figure S2C–E).

#### 3.7.2. Prevalence of *S. stercoralis* by Study Population (Age Group)

Among the included individual studies, 193 (57.4%), 13 (3.9%), and 10 (3.0%) were conducted specifically among school-aged children, women, and pre-school-aged children, respectively (Table 2). Twenty-eight (8.3%) of the included individual studies were pre-school-aged and school-based studies. Age categories of 81,559 participants were not mentioned. The highest *S. stercoralis* prevalence was 7.1% (95% CI: 6.1–8.1%, *I*^2^ = 90.4%, *p* < 0.001) and was reported in school-aged children, followed by 6.3% (95% CI: 3.7–8.9%, *I*^2^ = 83.0%, *p* < 0.001) reported in pre-school-aged children. In contrast, the lowest pooled *S. stercoralis* prevalence was of 1.6% (95% CI: 0.8–2.4%, *I*^2^ = 75.9%, *p* < 0.001) was obtained in individual studies which focused on female populations.

#### 3.7.3. Prevalence of *S. stercoralis* by Setting

Overall, 196 (58.3%) individual studies were carried out in schools. The remaining individual studies were either performed in communities (n = 98, 29.2%) or in hospitals (n = 42, 12.5%) (Table 1). The pooled *S. stercoralis* prevalence was 6.6% (95% CI: 5.7–7.5%, *I*^2^ = 98.3%, *p* < 0.001) in community-based studies, 5.5% (95% CI: 4.7–6.3%, *I*^2^ = 91.5%, *p* < 0.001) in school-based studies, and 1.5% (95% CI: 1.2–1.8%, *I*^2^ = 91.0%, *p* < 0.001) in hospital-based studies (Table 2). There were no significant differences in the pooled prevalence of *S. stercoralis* between the individual studies conducted in schools and those conducted in communities. In contrast, *S. stercoralis* prevalence in school-based and community-based studies were significantly higher compared to individual studies conducted in hospitals.

#### 3.7.4. Prevalence of *S. stercoralis* by Sample Size

There were 166 individual studies with a sample size of 100 participants or less (9111 individuals). In 144 individual studies the number of participants ranged between 101 and 1000 (45,977 individuals), 23 individual studies had a sample size of 1001 to 10,000 participants (42,661 individuals), and 3 individual studies included more than 10,000 participants each (45,363 individuals) (Table 2). The highest pooled *S. stercoralis* infection prevalence was found in individual studies with sample sizes lower than, or equal to, 100 participants (9.1%, 95% CI: 7.4–10.7%, *I*^2^ = 80.5%, *p* < 0.001) and the lowest prevalence of *S. stercoralis* was observed in individual studies with sample sizes ranging between 1001 and 10,000 participants (3.1%, 95% CI: 2.4–3.8%, *I*^2^ = 98.7, *p* < 0.001). The pooled *S. stercoralis* prevalence was significantly higher in study categories with sample sizes of 100 participants, or fewer, compared to individual studies with sample sizes higher than 100 participants.

#### 3.7.5. Prevalence of *S. stercoralis* by Gender

Among the included individual studies, 11 stratified *S. stercoralis* data by gender. Forest plots revealed that *S. stercoralis* prevalence was 4.7% (95% CI: 0.8–8.6%, *I*^2^ = 98.3%, *p* < 0.001) in females and 4.6% (95% CI: 0.3–8.8%, *I*^2^ = 98.6%, *p* < 0.001) in male participants (Figure S3). There was no statistical significant difference in prevalence of *S. stercoralis* by gender.

#### 3.7.6. Approaches for Strongyloidiasis Diagnosis

Table S4 summarises the characteristics of the diagnostic methods used in the included individual studies. Most individual studies employed KK (118, 35.1%), FE (79, 23.5%), and DA (23, 6.8%) for strongyloidiasis diagnosis. DA plus FE (22, 6.7%) was the combination of diagnostic tools frequently reported. The highest prevalence of *S. stercoralis* was 33.9% (95% CI: 24.8–43.1%) and obtained using a combination of FLOTAC, FE, KK, and KAP, followed by 33.3% (95% CI: 27.3–39.4%) recorded using a combination of KK and mertiolate iodine formaldehyde concentration (MIFC), and 33.0% (95% CI: 27.5–38.5%) using a combination of DA and merthiolate-formaldehyde concentration (MFC). When a combination of Baermann and KAP was used, the *S. stercoralis* prevalence was 18.8%. *S. stercoralis* prevalence was 12.6% using a combination of five diagnostic methods, namely Baermann, FLOTAC, FE, KK, and KAP. Among the individual studies that used only one diagnostic tool, the highest pooled strongyloidiasis prevalences were 17.1% (95% CI: 7.4–26.8%) observed by ELISA and 11.6% (95% CI: 11.2–12.1%) reported using coproculture (charcoal culture). The lowest pooled strongyloidiasis prevalence was 1.6% (95% CI: 0.5–2.7%), 2.5 (95% CI: 0.5–2.7%), 3.2% (95% CI: 1.1–5.3%), and 3.5% (95% CI: 2.8–4.1%) recorded using Ritchie, MBA, Roman, and KK technique, respectively.

### 3.8. Risk Factors for S. stercoralis Infection

**Figure 7:**
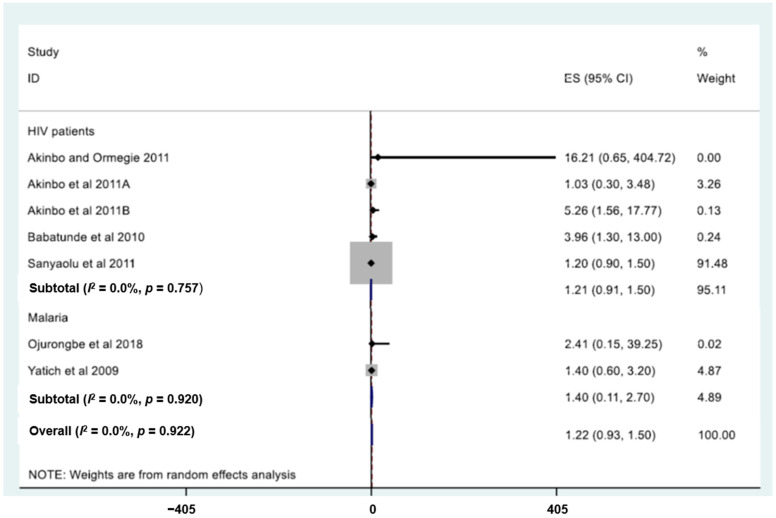
Forest plot showing the odds ratio of *Strongyloides stercoralis* infection among HIV and malaria patients in West Africa [69,85,86,87,88,89,133]. Grey square represents the weight of each study in the meta-analysis. The box shows the odds ratio of each study. The vertical line corresponds to no effect.

Table 5 summarises the characteristics of risk factors for *S. stercoralis* infection in West Africa. Twelve individual studies were included to determine the risk factors for *S. stercoralis* infection. There were five types of predictors: diseases (HIV and malaria), symptoms (anaemia, stomach ache, and nausea), water, sanitation, and hygiene indicators (WASH, e.g., use of community tap water, availability of water or tissue for use after defaecation, availability of water and/soap after defaecation), demographic parameters (gender and age categories), and intervention. HIV (n = 5) was the main *S. stercoralis* infection predictor reported, followed by malaria (two studies) (Figure 7). The odds of *S. stercoralis* infection was higher among HIV patients compared to HIV-negative people (OR = 1.2, 95% CI: 0.9–1.5). However, there was no significant difference in pooled prevalence of *S. stercoralis* between HIV patients compared to HIV-negative people. Malaria patients were at higher odds of being infected with *S. stercoralis* larvae than malaria-negative individuals (OR = 1.4, 95% CI: 0.1–2.7), but there was no significant difference in pooled prevalence of *S. stercoralis* between malaria patients and their counterparts without the disease.

## 4. Discussion

Strongyloidiasis is an underappreciated parasitic worm infection, which can be partially explained by the challenge of accurate diagnosis, particularly in resource-constrained settings [1,150]. As a consequence, strongyloidiasis is usually not included in control programmes, although the combination of albendazole and ivermectin used for lymphatic filariasis and soil-transmitted helminthiasis also impacts strongyloidiasis [151,152]. The endemicity of strongyloidiasis is unknown across large parts of West Africa. The aim of the systematic review and meta-analysis presented here was to fill an important gap by providing the pooled *S. stercoralis* prevalence. We found an overall pooled *S. stercoralis* prevalence of slightly lower than 5% with considerable heterogeneity (*I*^2^ = 96.8%). This result is in line with studies conducted among children in urban settings in Côte d’Ivoire [33,36,44], Nigeria [96], and Senegal [145]. In contrast, our estimate was considerably lower than the overall prevalence of *S. stercoralis* reported in rural areas among schoolchildren in south-central and south-eastern parts of Côte d’Ivoire (27.0%) [39]. Similarly, a considerably higher prevalence of *S. stercoralis* infection (64.5%) was observed in a study carried out in rural populations of two regions in central and southern Togo [15]. One plausible explanation of the relative lower prevalence of *S. stercoralis* in West African countries might be the main objective and diagnostic methods of the studies. Among the included studies in this review, less than 10% were specifically designed to study the epidemiology of *S. stercoralis*. Hence, most of the diagnostic methods utilised in these studies only had a low sensitivity for *S. stercoralis* diagnosis, such as KK and FE methods, resulting in very low *S. stercoralis* detection rate. The observed strongyloidiasis cases were mainly accidental. The reported high *S. stercoralis* prevalence in some studies could be due to the characteristics of the study areas and the diagnostic methods used. Indeed, the studies in the two regions of Côte d’Ivoire and Togo were carried out in rural areas which provided suitable conditions for *S. stercoralis* transmission. In addition, sensitive *S. stercoralis* diagnostic tools, such as Baermann, KAP, and ELISA were used leading to the relatively high *Strongyloides* prevalence [38,153].

Our findings revealed that in West Africa the prevalence of *S. stercoralis* varied considerably, from less than 1% in Burkina Faso to 18.9% in The Gambia. This observation might be due to characteristics of the study population, environmental features, and socioeconomic status, which vary between, and within, countries. In Nigeria, where the highest number of the included studies was reported, the prevalence of *S. stercoralis* was 5.1% and was similar to the result (4.9%) of a recent systematic review and meta-analysis on *S. stercoralis* prevalence, diagnostic methods, and settings in Africa [10]. In Côte d’Ivoire, where the third highest number of studies (n = 46) included in our meta-analysis, the pooled prevalence of *S. stercoralis* was approximately the same (5.2%), which is significantly lower than 24.5% presented in a recent review [10]. The difference in the prevalence of *S. stercoralis* in Côte d’Ivoire may be due to the number of eligible studies and diagnostic methods used. Indeed, in the aforementioned review, two studies using the PCR technique were included, while in the current systematic review, microscopy-based techniques were predominantly utilised for diagnosis. It is widely acknowledged that DNA-based techniques are more sensitive than parasitological approaches [154,155]. Hence, the studies using DNA-based techniques produced higher prevalence of *S. stercoralis* infection than the parasitological studies.

Our findings revealed that considering different diagnostic tools, charcoal culture was more sensitive to the detection of *S. stercoralis* than the Baermann method. However, previous studies showed that charcoal culture was less sensitive than the Baermann method [156]. The relatively higher *S. stercoralis* prevalence using charcoal culture found in the current review might be due to the very high sample size (20,250 individuals) in the study carried out in the northern part of Ghana [134].

The results of the current review showed that the prevalence of *S. stercoralis* was lower when using the commonly employed Baermann method (5.1%). This observation corroborates observations from the south-central part of Côte d’Ivoire (8.2%) and the south-western part of Angola [31,157]. The findings indicated that the association of Baermann with KAP increased the estimated prevalence of *S. stercoralis* three-fold. These findings suggest that although the combination of diagnostic methods can lead to an increase in the prevalence of *S. stercoralis* [10,158,159], several other components of the association, such as sample size, characteristics of the study population, and environmental parameters might significantly influence the occurrence of *S. stercoralis* infection.

Considering one diagnostic technique, the highest prevalence of *S. stercoralis* infection was found using the ELISA technique. This observation confirms that the ELISA technique is a promising tool for *S. stercoralis* diagnosis. However, the limitation of the ELISA technique is the cross-reactions with other helminths which might overestimate the prevalence of *S. stercoralis* infection [160].

Our systematic review, including 336 individual studies, indicated that the magnitude of the pooled *S. stercoralis* prevalence in West Africa decreased significantly from 10.9% in 1975–1984 to 4.3% in 2015–2024. The decreasing trend could be attributed to social and economic development, and the establishment and long-term monitoring of onchocerciasis control programmes based on ivermectin treatment over large parts of West Africa, starting in the 1990s [161]. Another reason for the reduction in the pooled prevalence of *S. stercoralis* infection could be attributed to the implementation of the London Declaration by governments and non-governmental organisations who support the control of helminthiasis and other neglected tropical diseases through large-scale mass drug administrations and Vitamin A supplementation campaigns using albendazole tablets [162,163].

Our findings revealed that the highest *S. stercoralis* prevalences were found among school-aged children, followed by pre-school-aged children. Hence, the strongyloidiasis programmes should target pre-school- and school-aged children for the control of the disease. In addition, adult population at high risk, such as pregnant women and immunosuppressed individuals, should also be targeted during treatment campaigns. In addition, adult populations are barely covered by studies conducted thus far. Hence, it would be biologically plausible that the prevalence rises with age.

The results of the current review revealed that the odds of *S. stercoralis* infection was not significantly different among HIV patients and malaria-positive people compared to healthy individuals. These findings differ with results of studies among HIV-positive people and pregnant women in Ghana and Nigeria [88,89]. The finding in the current review might be due to the characteristics and limited number of the studies. Prior observations suggest that HIV and malaria patients are particularly vulnerable to *S. stercoralis* infection [69,85]. These infectious diseases might increase the risk of strongyloidiasis. Thus, it is important to examine all patients with HIV and malaria for concurrent *S. stercoralis* infection.

### Strengths and Limitations

A limitation of the current review is the use of only online databases, coupled with a large variety of diagnostic tools in different social-ecological settings. Another limitation is the predominance of studies that were not primarily focused on *Strongyloides*, and hence, lacked data on specific high-risk populations such as adults, pregnant women, people with HIV, or malaria patients. However, the use of eight readily available databases and the inclusion of studies published in the past 50 years, regardless of language, increased considerably the sensitivity of the search strategy and resulted in strong evidence of the provided data. Of note, our data will become available via the Global Neglected Tropical Diseases (GNTD) database for decision-makers and other stakeholders dedicated to the epidemiology and control of strongyloidiasis and other neglected tropical diseases.

## 5. Conclusions

The current review revealed that *S. stercoralis* infection is reported in all but one of the 16 West African countries. *S. stercoralis* infection prevalence did not differ between pre-school-aged and school-aged children. These findings confirm the importance of including strongyloidiasis along soil-transmitted helminthiasis control programmes and to consider pre-school-aged children in targeted populations in the control strategies. Parasitological tools commonly used as diagnostic techniques have low sensitivity for *S. stercoralis* diagnosis. Hence, the reported *S. stercoralis* prevalence is likely an underestimation of the true situation. This observation calls for more sensitive diagnostic tools for an accurate estimation of the true strongyloidiasis distribution and public health problem. We recommend governments of West Africa to promote WASH, coupled with setting-specific information, education, and communication strategies, in order to lower the burden of strongyloidiasis. The highest prevalences of *S. stercoralis* infection was observed in the Gulf of Guinea. Future research should assess the prevalence and intensity of *S. stercoralis* infection using sensitive diagnostic approaches and determine associated risk factors. Particular emphasis should be placed on the impact of the geographic parameters on the disease burden in the context of climate change.

## Figures and Tables

**Table 1:** Demographic characteristics of the individual studies stratified by West African countries.

Country	N Individual Studies	Study Setting			Study Population					References
		School	Hospital	Community	All Age and Sex	PSAC	SAC	PSAC + SAC	Females	
The Gambia	19	18	0	1	0	0	19	0	0	Kositz et al. (2022) [12], Sanyang et al. (2021) [13]
Liberia	2	2	0	0	0	0	2	0	0	Sodeman (1979) [14]
Togo	10	1	1	8	7	2	1	0	0	Korbmacher et al. (2018) [15], Gbadoé et al. (2005) [16], Aplogan et al. (1990) [17], Lapierre et al. (1988) [18]
Guinea-Bissau	5	1	1	3	1	1	1	2	0	Farrant et al. (2020) [19], von Huth et al. (2019) [20], Steenhard et al. (2009) [21], Lebbad et al. (2001) [22], Carstensen et al. (1987) [23]
Sierra Leone	3	0	0	3	3	0	0	0	0	Bailey et al. (2006) [24], Gbakima (1994) [25], Whitworth et al. (1991) [26]
Cape Verde	1	0	1	0	0	0	0	1	0	Colito et al. (2021) [27]
Guinea	10	0	0	10	0	0	8	2	0	Beavogui et al. (2021) [28], Glickman et al. (1999) [29], Gyorkos et al. (1996) [30]
Côte d’Ivoire	46	36	1	9	5	1	25	15	0	Becker et al. (2015b) [31], Becker et al. (2015a) [32], Adoubryn et al. (2012) [33], Becker et al. (2011) [34], Traoré et al. (2011) [35], Djohan et al. (2010) [36], Diakité et al. (2010) [37], Glinz et al. (2010b) [38], Glinz et al. (2010a) [39], Menan et al. (2008) [40], Evi et al. (2007) [41], Yapi et al. (2006) [42], Dancesco et al. (2005) [43], Agbaya et al. (2004) [44], Menan et al. (1997b) [45], Menan et al. (1997a) [46], Therizol-Ferly et al. (1989) [47], Penali et al. (1988) [48], Haller and Lauber (1980) [49], Nozais et al. (1975) [50]
Nigeria	119	69	25	25	41	1	66	7	4	Gbonhinbor et al. (2024) [51], Agabi et al. (2023) [52], Alade et al. (2023) [53], Imalele et al. (2023) [54], Udeh et al. (2023) [55], Evbuomwan et al. (2022) [56], Inah et al. (2022) [57], Nwankwo et al. (2021) [58], Yaro et al. (2021) [59], Nnolim et al. (2020) [60], Abe et al. (2019) [61], Dahal et al. (2019) [62], Dawaki et al. (2019) [63], Udeh et al. (2019) [64], Ayede et al. (2018) [65], Akinbo and Akanbi (2018) [66], Amoo et al. (2018) [67], Gbebi et al. (2018) [68], Ojurongbe et al. (2018) [69], Adeniran et al. (2017) [70], Inyang-Etoh et al. (2017) [71], Ivoke et al. (2017) [72], Muazu et al. (2017) [73], Shitta and Akogun (2017) [74], Taiwo et al. (2017) [75], Douglas and Amadi (2016) [76], Obateru et al. (2016) [77], Abah and Arene (2015) [78], Agu et al. (2013) [79], Ohaeri and Orji (2013) [80], Abaver et al. (2012) [81], Akinbo et al. (2012) [82], Oguanya et al. (2012) [83], Abaver et al. (2011) [84], Akinbo et al. (2011b) [85], Akinbo et al. (2011a) [86], Akinbo and Ormegie (2011) [87], Sanyaolu et al. (2011) [88], Babatunde et al. (2010) [89], Dada-Adegbola et al. (2010) [90], Damen et al. (2010) [91], Umar and Bassey (2010) [92], Houmsou et al. (2009) [93], Morenikeji et al. (2009) [94], Ibidapo and Okwa (2008) [95], Adeoye et al. (2007) [96], Agbolade et al. (2007) [97], Ikeh et al. (2007) [98], Ugbomoiko and Ofoezie (2007) [99], Wagbatsoma and Aisien (2005) [100], Anosike et al. (2004) [101], Dada-Adegbola and Bakare (2004) [102], Anyaeze (2003) [103], Okoronkwo (2002) [104], Ozumba and Ozumba (2002) [105], Nwokediuko et al. (2002) [106], Nwaorgu et al. (1998) [107], Sodipo et al. (1997) [108], Udonsi et al. (1996) [109], Asaolu et al. (1992) [110], Akogun (1989) [111], Holland et al. (1989) [112], Arene (1984) [113]
Ghana	28	2	5	21	13	5	3	1	6	Backhaus et al. (2024) [114], Deku et al. (2024) [115], Donkoh et al. (2023) [116], Akenten et al. (2022) [117], Fornace et al. (2022) [118], Aninagyei et al. (2020) [119], Cunningham et al. (2020) [120], Kpene et al. (2020) [121], Adu-Gyasi et al. (2018) [122], Cunningham et al. (2018) [123], Forson et al. (2017) [124], Tay et al. (2017) [125], Agboli et al. (2015) [126], Danikuu et al. (2015) [127], Duedu et al. (2015) [128], Tay et al. (2011) [129], Baidoo et al. (2010) [130], Yatich et al. (2010) [131], Verweij et al. (2009) [132], Yatich et al. (2009) [133], Yelifari et al. (2005) [134], Annan et al. (1986) [135]
Benin	67	66	0	1	1	0	66	0	0	Boko et al. (2016) [136], Ibikounlé et al. (2014) [137], Chippaux et al. (1990) [138]
Mauritania	16	1	0	15	14	0	2	0	0	Ba et al. (2024) [139], Urbani et al. (1997) [140], WHO (1995) [141]
Senegal	4	0	3	1	4	0	0	0	0	Diongue et al. (2017) [142], Sow et al. (2017) [143], Ndiaye et al. (2013) [144], Salem et al. (1994) [145]
Niger	1	0	0	1	1	0	0	0	0	Mouchet et al. (1988) [146]
Burkina Faso	5	0	5	0	2	0	0	0	3	Sangaré et al. (2021) [147], Sangaré et al. (2015) [148], Karou et al. (2011) [149]
Mali	…	No data	…	…	…	…	…	…	…	
Total	336	196	42	98	92	10	193	28	13	

**Table 2:** Pooled prevalence of *Strongyloides stercoralis* infection in West Africa by demographic characteristics.

Variable		Sample Size	No Positive	Pooled Prevalence (%)	95% CI	*I*^2^ (%)	*p*-Value
Population							
	All age and sex	85,082	4333	4.9	4.2–5.5	98.3	<0.001
	PSAC	1927	127	6.3	3.7–8.9	83.0	<0.001
	SAC	31,376	1469	7.1	6.1–8.1	90.6	<0.001
	PSAC + SAC	19,955	324	2.0	1.4–2.5	90.3	<0.001
	Females	4772	91	1.6	0.8–2.4	75.9	<0.001
Study setting							
	School	34,582	1325	5.5	4.7–6.3	91.5	<0.001
	Hospital	49,266	629	1.5	1.2–1.8	91.0	<0.001
	Community	59,264	4390	6.6	5.7–7.5	98.3	<0.001
Sample size							
	<101	9111	544	9.1	7.4–10.7	80.5	<0.001
	101–1000	45,977	1977	4.7	4.1–5.3	93.1	<0.001
	1001–10,000	42,661	1335	3.1	2.4–3.8	98.7	<0.001
	>10,000	45,363	2488	4.2	0.7–7.7	99.9	<0.001

CI: confidence interval; PSAC: pre-school-aged children; SAC: school-aged children.

**Table 3:** Meta-regression analysis of *Strongyloides stercoralis* prevalence in West Africa from 1975 to 2024.

Variable	β-Coefficient	95% CI	*p*-Value
Publication decade	0.90	0.77–1.04	0.141
Study population	0.95	0.82–1.10	0.478
Study country	1.01	0.95–1.05	0.812
Study setting	0.92	0.75–1.12	0.433
Diagnostic method	0.99	0.97–1.01	0.540
Sample size	0.42	0.34–0.53	<0.001

**Table 4:** Prevalence of *Strongyloides stercoralis* infection stratified by West African countries.

Country	N Individual Studies	N Participants	N Positive	*S. stercoralis* Prevalence				References
				(%)	95% CI	*I* ^2^	*p*-Value	
The Gambia	19	1859	203	18.9	11.1–26.8	88.4	<0.001	Kositz et al. (2022) [12], Sanyang et al. (2021) [13]
Liberia	2	549	84	14.9	9.8–19.9	62.3	0.103	Sodeman (1979) [14]
Togo	10	1749	149	10.7	6.9–14.6	92.3	<0.001	Korbmacher et al. (2018) [15], Gbadoé et al. (2005) [16], Aplogan et al. (1990) [17], Lapierre et al. (1988) [18]
Guinea-Bissau	5	2631	126	7.8	2.4–13.2	96.4	<0.001	Farrant et al. (2020) [19], von Huth et al. (2019) [20], Steenhard et al. (2009) [21], Lebbad et al. (2001) [22], Carstensen et al. (1987) [23]
Sierra Leone	3	2076	145	6.9	5.5–8.3	20.7	0.283	Bailey et al. (2006) [24], Gbakima (1994) [25], Whitworth et al. (1991) [26]
Cape Verde	1	105	7	6.8	2.7–13.2	…	…	Colito et al. (2021) [27]
Guinea	10	1478	84	5.5	2.8–8.1	80.8	<0.001	Beavogui et al. (2021) [28], Glickman et al. (1999) [29], Gyorkos et al. (1996) [30]
Côte d’Ivoire	46	16,119	464	5.2	4.1–6.2	92	<0.001	Becker et al. (2015b) [31], Becker et al. (2015a) [32], Adoubryn et al. (2012) [33], Becker et al. (2011) [34], Traoré et al. (2011) [35], Djohan et al. (2010) [36], Diakité et al. (2010) [37], Glinz et al. (2010b) [38], Glinz et al. (2010a) [39], Menan et al. (2008) [40], Evi et al. (2007) [41], Yapi et al. (2006) [42], Dancesco et al. (2005) [43], Agbaya et al. (2004) [44], Menan et al. (1997b) [45], Menan et al. (1997a) [46], Therizol-Ferly et al. (1989) [47], Penali et al. (1988) [48], Haller and Lauber (1980) [49], Nozais et al. (1975) [50]
Nigeria	119	47,705	2106	5.1	4.4–5.9	96.6	<0.001	Gbonhinbor et al. (2024) [51], Agabi et al. (2023) [52], Alade et al. (2023) [53], Imalele et al. (2023) [54], Udeh et al. (2023) [55], Evbuomwan et al. (2022) [56], Inah et al. (2022) [57], Nwankwo et al. (2021) [58], Yaro et al. (2021) [59], Nnolim et al. (2020) [60], Abe et al. (2019) [61], Dahal et al. (2019) [62], Dawaki et al. (2019) [63], Udeh et al. (2019) [64], Ayede et al. (2018) [65], Akinbo and Akanbi (2018) [66], Amoo et al. (2018) [67], Gbebi et al. (2018) [68], Ojurongbe et al. (2018) [69], Adeniran et al. (2017) [70], Inyang-Etoh et al. (2017) [71], Ivoke et al. (2017) [72], Muazu et al. (2017) [73], Shitta and Akogun (2017) [74], Taiwo et al. (2017) [75], Douglas and Amadi (2016) [76], Obateru et al. (2016) [77], Abah and Arene (2015) [78], Agu et al. (2013) [79], Ohaeri and Orji (2013) [80], Abaver et al. (2012) [81], Akinbo et al. (2012) [82], Oguanya et al. (2012) [83], Abaver et al. (2011) [84], Akinbo et al. (2011b) [85], Akinbo et al. (2011a) [86], Akinbo and Ormegie (2011) [87], Sanyaolu et al. (2011) [88], Babatunde et al. (2010) [89], Dada-Adegbola et al. (2010) [90], Damen et al. (2010) [91], Umar and Bassey (2010) [92], Houmsou et al. (2009) [93], Morenikeji et al. (2009) [94], Ibidapo and Okwa (2008) [95], Adeoye et al. (2007) [96], Agbolade et al. (2007) [97], Ikeh et al. (2007) [98], Ugbomoiko and Ofoezie (2007) [99], Wagbatsoma and Aisien (2005) [100], Anosike et al. (2004) [101], Dada-Adegbola and Bakare (2004) [102], Anyaeze (2003) [103], Okoronkwo (2002) [104], Ozumba and Ozumba (2002) [105], Nwokediuko et al. (2002) [106], Nwaorgu et al. (1998) [107], Sodipo et al. (1997) [108], Udonsi et al. (1996) [109], Asaolu et al. (1992) [110], Akogun (1989) [111], Holland et al. (1989) [112], Arene (1984) [113]
Ghana	28	36,854	2671	3.7	2.0–5.4	98.9	<0.001	Backhaus et al. (2024) [114], Deku et al. (2024) [115], Donkoh et al. (2023) [116], Akenten et al. (2022) [117], Fornace et al. (2022) [118], Aninagyei et al. (2020) [119], Cunningham et al. (2020) [120], Kpene et al. (2020) [121], Adu-Gyasi et al. (2018) [122], Cunningham et al. (2018) [123], Forson et al. (2017) [124], Tay et al. (2017) [125], Agboli et al. (2015) [126], Danikuu et al. (2015) [127], Duedu et al. (2015) [128], Tay et al. (2011) [129], Baidoo et al. (2010) [130], Yatich et al. (2010) [131], Verweij et al. (2009) [132], Yatich et al. (2009) [133], Yelifari et al. (2005) [134], Annan et al. (1986) [135]
Benin	67	10,583	87	3.5	2.1–5.0	70.3	<0.001	Boko et al. (2016) [136], Ibikounlé et al. (2014) [137], Chippaux et al. (1990) [138]
Mauritania	16	2503	72	2.9	1.2–4.6	68.7	0.001	Ba et al. (2024) [139], Urbani et al. (1997) [140], WHO (1995) [141]
Senegal	4	4772	119	2.8	0.0–5.9	97.0	<0.001	Diongue et al. (2017) [142], Sow et al. (2017) [143], Ndiaye et al. (2013) [144], Salem et al. (1994) [145]
Niger	1	1900	6	0.3	0.1–0.7	…	…	Mouchet et al. (1988) [146]
Burkina Faso	5	12,229	21	0.2	0.1–0.2	0	0.758	Sangaré et al. (2021) [147], Sangaré et al. (2015) [148], Karou et al. (2011) [149]
Mali	…	…	…	…	…	…	…	
Total	336	143,112	6344	4.4	4.1–4.8	96.8	<0.001	

CI: confidence interval; PSAC: pre-school-aged children; SAC: school-aged children.

**Table 5:** Risk factors for *Strongyloides stercoralis* infection in West Africa, as revealed by a systematic review covering a 50-year period (1975 to 2024).

Type of Predictor	Variable	OR	95% CI	*p*-Value	Reference
Disease	HIV patients	16.2	0.7–404.7	0.347	[87]
	HIV patients	1.0	3.5	0.966	[86]
	HIV patients	5.3	1.6–17.8	0.006	[85]
	HIV patients	4.0	1.3–13.0	0.010	[89]
	HIV patients	1.2	0.9–1.5	0.067	[88]
	Malaria	2.4	0.2–39.3	0.500	[69]
	Malaria	1.4	0.6–3.2	NA	[133]
Symptoms	Anaemia	3.4	0.2–56.8	0.383	[86]
	Anaemia	1.7		0.130	[19]
	Stomach ache	2.4	1.0–5.6	0.056	[34]
	Nausea	5.0	1.3–19.3	NA	[32]
WASH indicators	Use of community tap water	6.2	1.4–28.2	0.019	[34]
	Water or tissue available sometime	1.4	0.8–2.5	0.080	[13]
	Water and soap available	0.9	0.5–1.6	0.613	[13]
Demography	Pre-school-aged children	0.3		<0.010	[19]
	7–10 years	3.3	1.8–5.8	<0.001	[13]
Intervention	Females	1.1	0.7–1.7	0.818	[13]
	MDA	0.4	0.2–1.0	0.037	[12]

CI: confidence interval; MDA: mass drug administration; OR: odds ratio; WASH: water, sanitation, and hygiene.

## Data Availability

Data used in this systematic review and meta-analysis will be freely available on Global Neglected Tropical Diseases website (www.gntd.org).

## Supplementary Materials

The following supporting information can be downloaded at: https://www.mdpi.com/article/10.3390/tropicalmed10110321/s1, Figure S1A: Funnel plot for the prevalence of *S. stercoralis* infection; Egger’s test (*p* < 0.001); Figure S1B: Meta-regression plot of categories of sample size (*p* < 0.001); Figure S2. Prevalence of *S. stercoralis* infection in West Africa (A: from 1975 to 1984, B: from 1985 to 1994); Figure S2C. Prevalence of *S. stercoralis* infection in West Africa between 1995 and 2004; Figure S2D. Prevalence *of S. stercoralis* infection in West Africa between 2005 and 2014; Figure S2E. Prevalence of *S. stercoralis* infection in West Africa between 2015 and 2024; Figure S3. Prevalence of *S. stercoralis* infection in West Africa by sex (A: Prevalence of *S. stercoralis* in males, B: Prevalence of *S. stercoralis* in females); Table S1: The PRISMA Group (2009). Preferred Reporting Items for Systematic Reviews and Meta-Analyses; Table S2: Characteristics of the included studies; Table S3: Quality assessment of the included studies; Table S4. Pooled prevalence of *S. stercoralis* infection in West Africa by diagnostic techniques.
