# Supplementary material for: Strongyloides stercoralis Infection in Humans in West Africa, 1975–2024: Systematic Review and Meta-Analysis
Source: Trop Med Infect Dis. 2025 Nov 17;10(11):321. doi: 10.3390/tropicalmed10110321 (PMC12656624; doi:10.3390/tropicalmed10110321)
Supplement: Supplementary file 1 [file tropicalmed-10-00321-s001.zip › Figure S1S2S3_Table S4_.pdf]

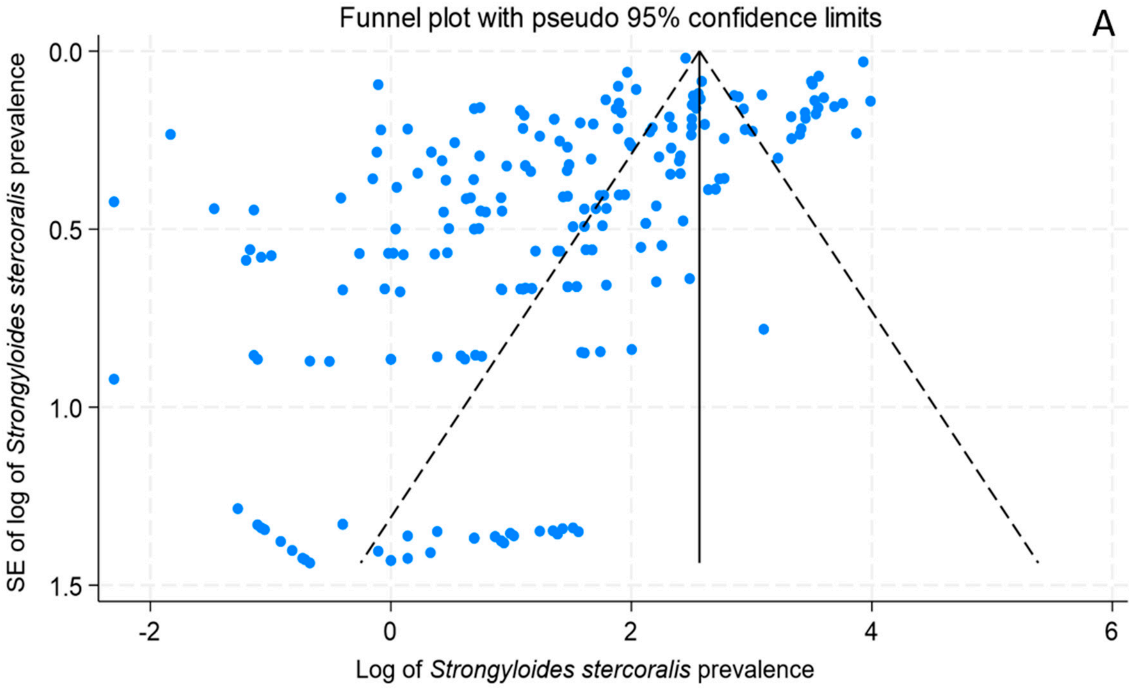

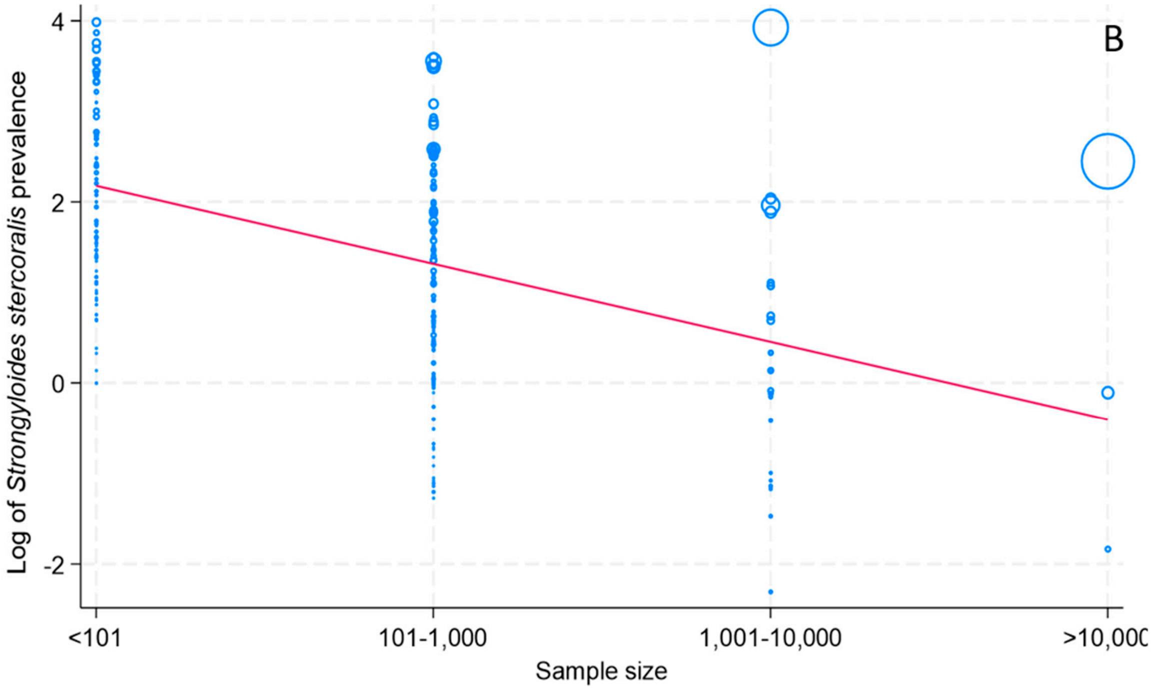

Figure S1. Funnel plot and meta-regression analysis for *Strongyloides stercoralis* infection studies in West Africa

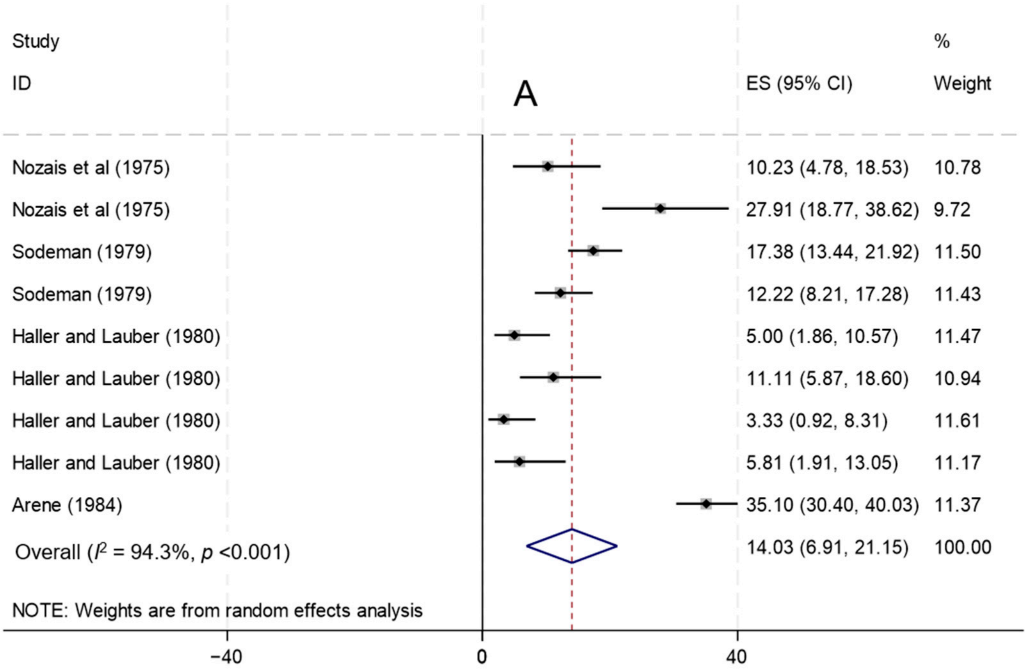

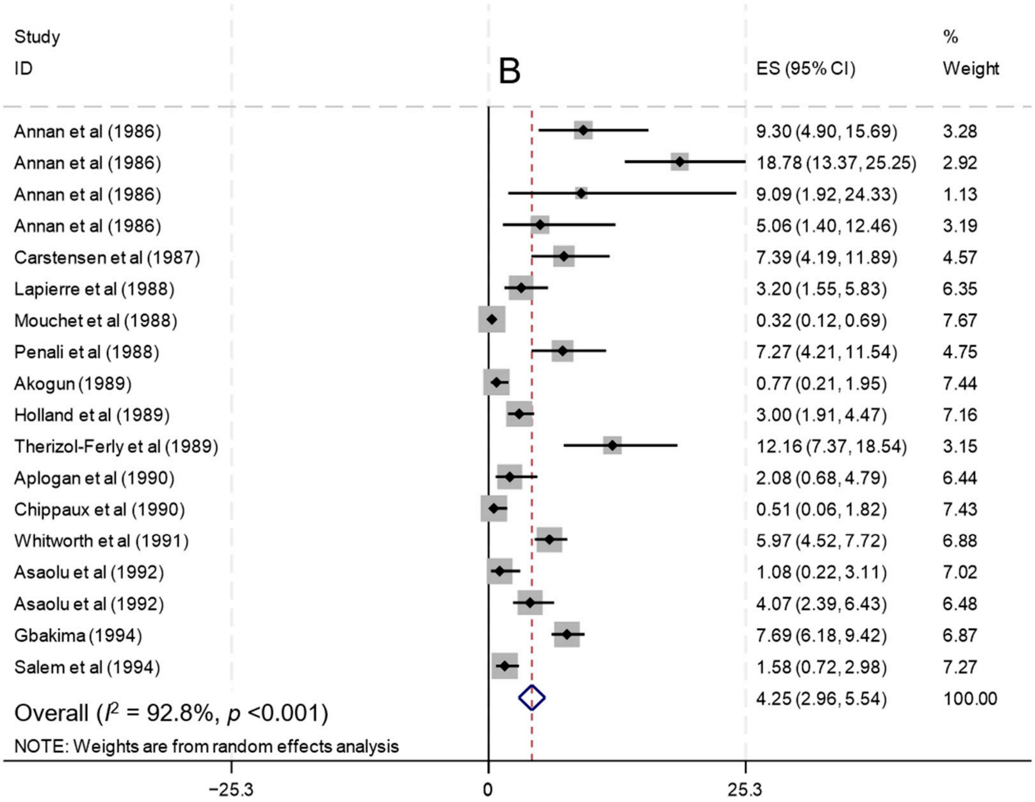

Figure S2. Prevalence of *S. stercoralis* infection in West Africa [14, 17, 18, 23, 25, 26, 47–50, 110, 112, 119, 135, 138, 145, 146]

A: from 1975 to 1984, B: from 1985 to 1994

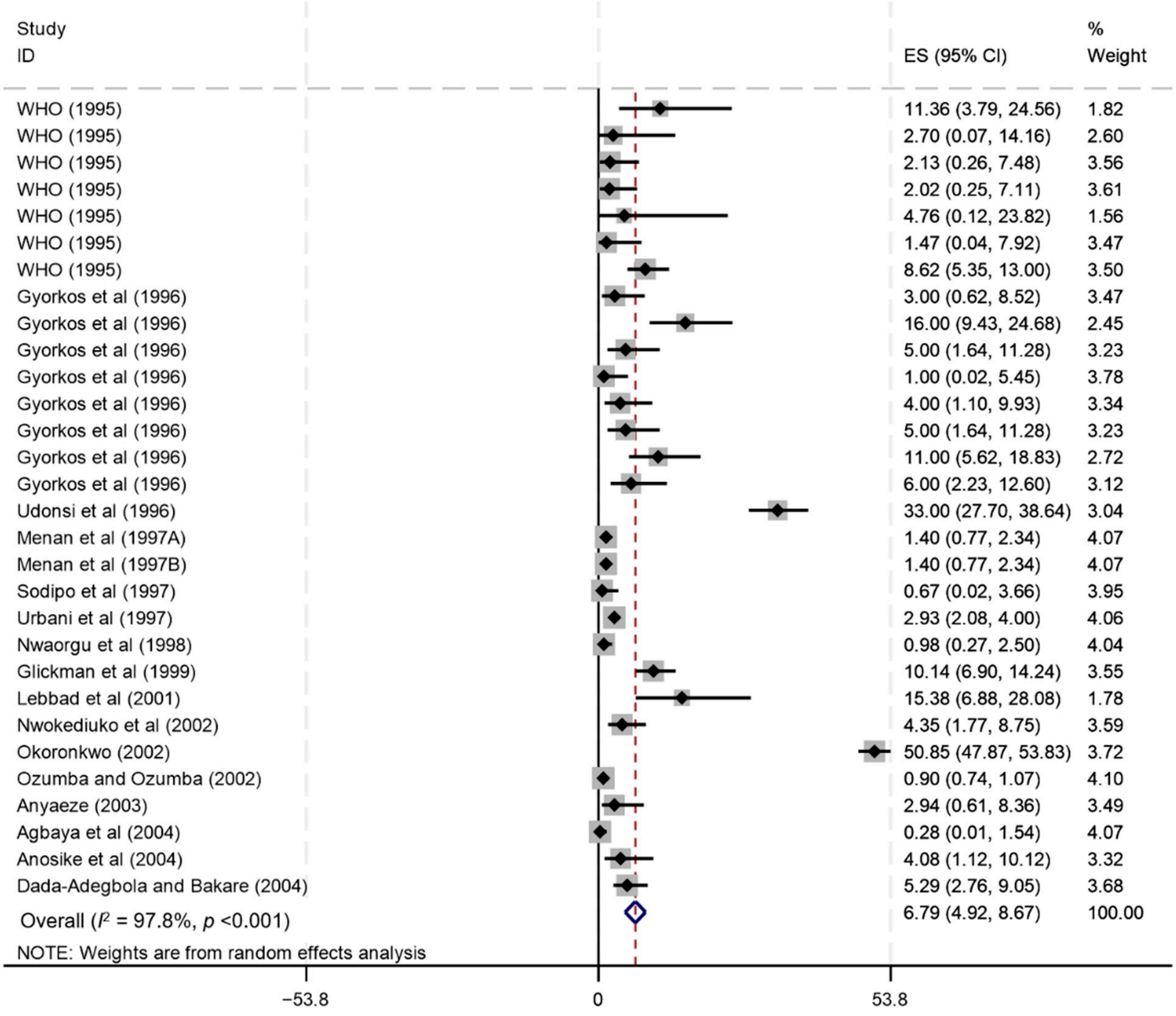

Figure S2 C. Prevalence of *S. stercoralis* infection in West Africa between 1995 and 2004 [22, 29, 30, 44–46, 90, 101, 103–109, 140, 141]

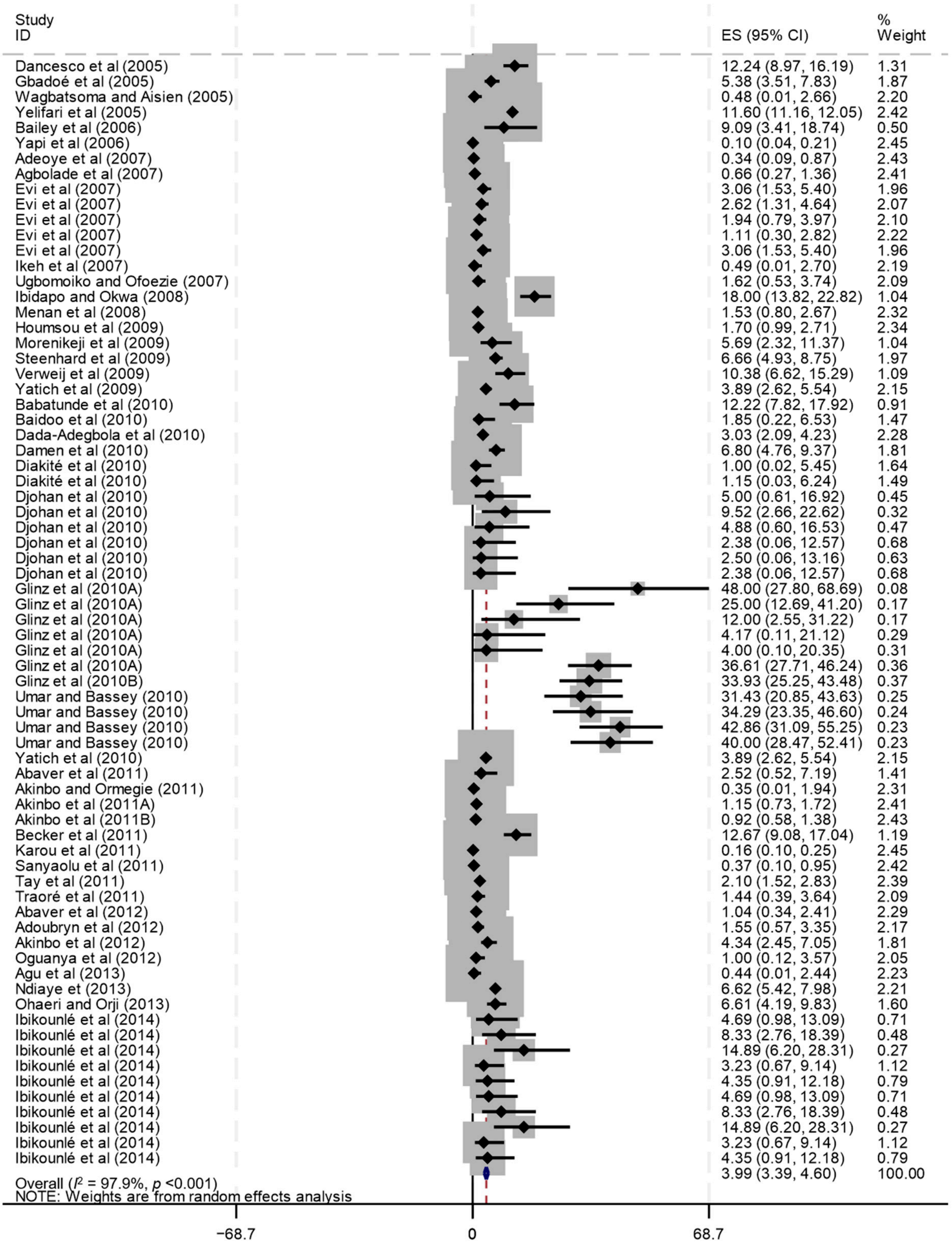

Figure S2 D. Prevalence of *S. stercoralis* infection in West Africa between 2005 and 2014 [16, 21, 24, 33–43, 79–97, 99, 100, 129–134, 137, 144, 149]

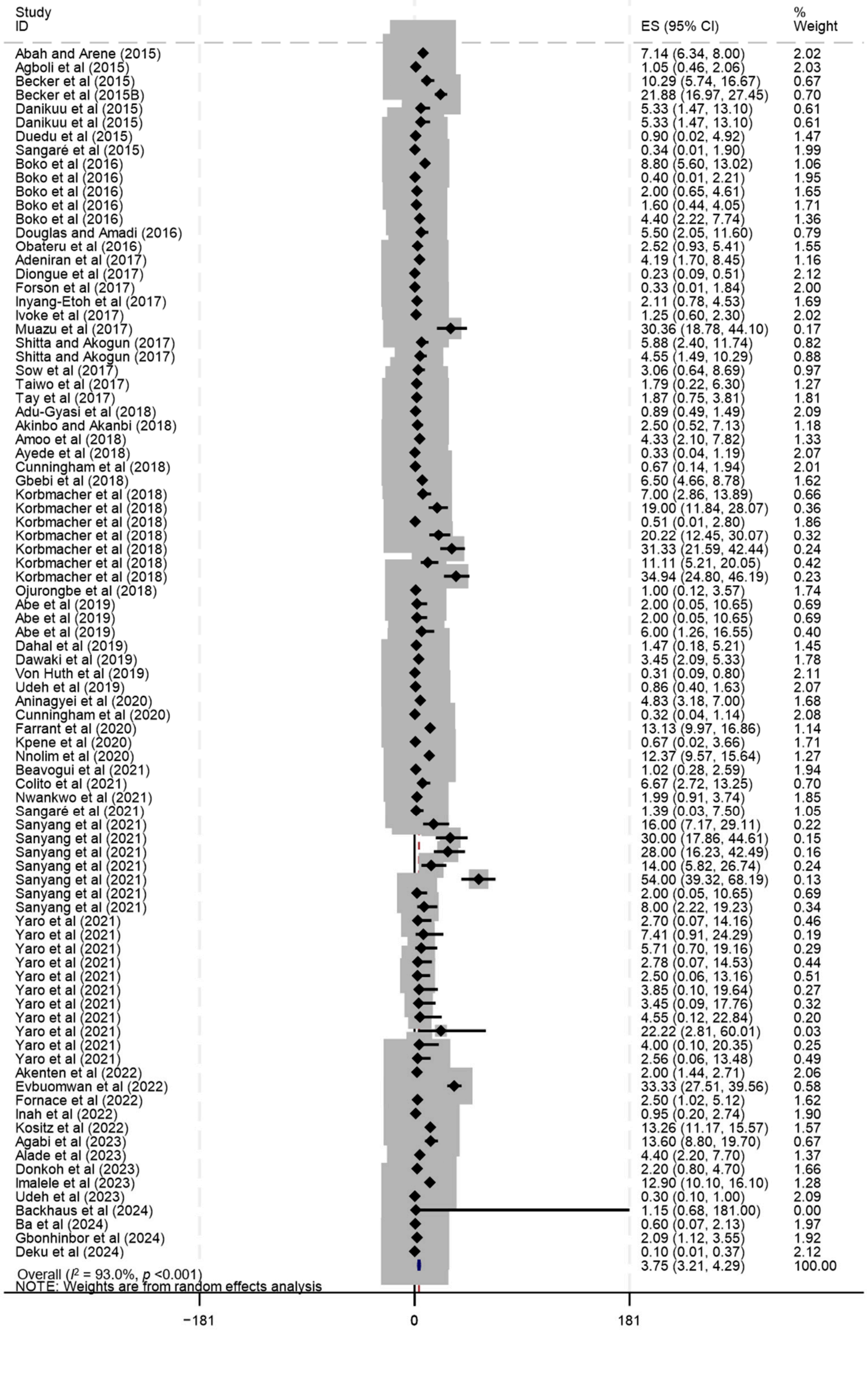

Figure S2 E. Prevalence of *S. stercoralis* infection in West Africa between 2015 and 2024 [12, 13, 15 19, 20, 27, 28, 31, 32, 51–78, 114–128, 136, 139, 142, 143, 147, 148]

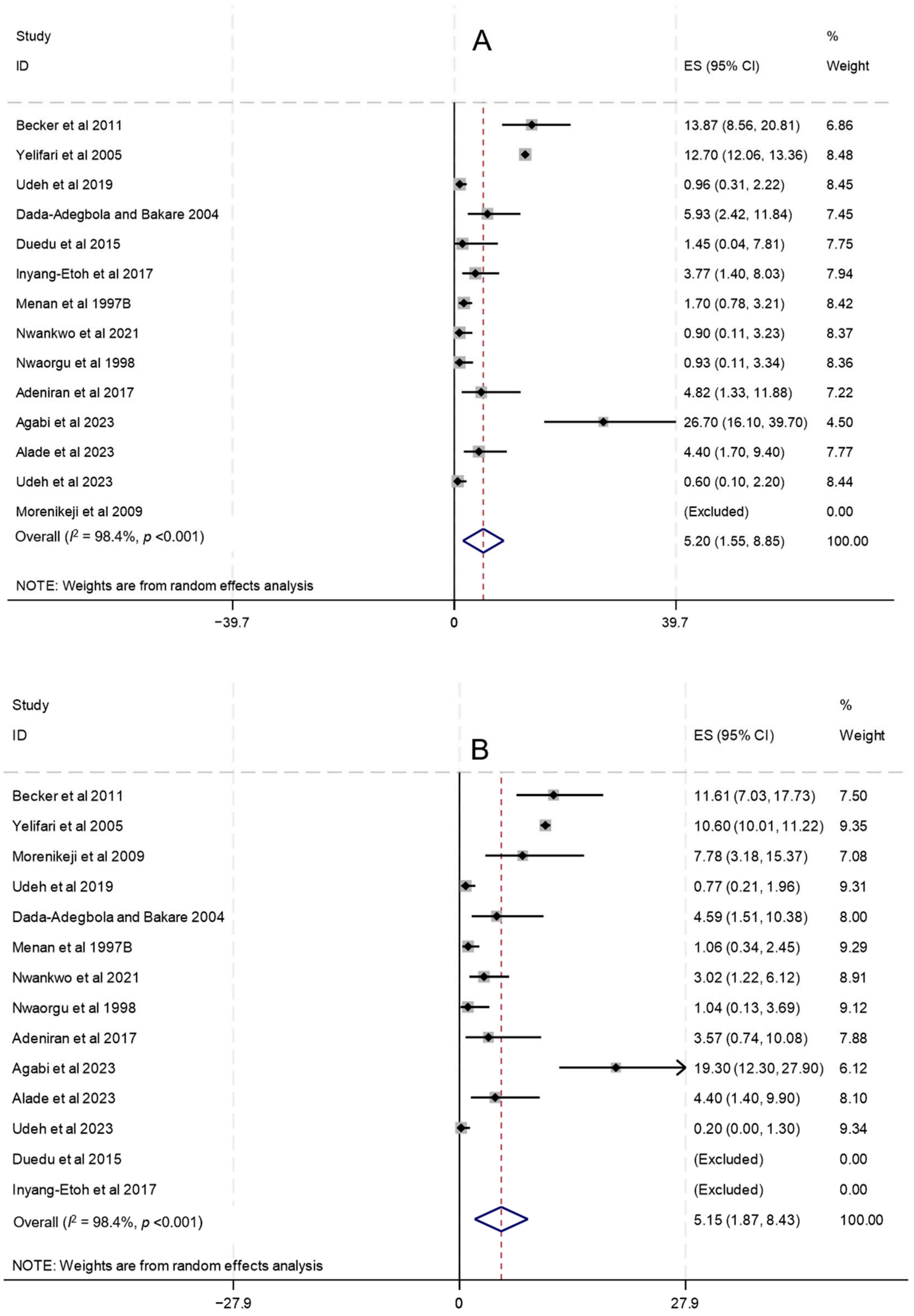

Figure S3. Prevalence of *Strongyloides* infection in West Africa by sex [34, 45, 52, 53, 55, 58, 64, 70, 71, 90, 94, 107, 128, 134]

A: Prevalence of *Strongyloides* in males, B: Prevalence of *Strongyloides* in females

Table S4. Pooled prevalence of *Strongyloides* infection in West Africa by diagnostic techniques

Diagnostic method	No of articles	Sample size	No positive	Pooled prevalence (%)	95% CI	I^2 (%)	p-value
Baermann	3	1,704	80	5.1	2.7-7.4	75.5	<0.001
Coproculture	2	20,316	2,355	11.6	11.2-12.0	0.0	<0.001
DA	23	19,247	520	6.6	5.4-7.8	96.4	<0.001
ELISA	7	733	109	17.1	7.4-26.8	94.6	p = 0.001
FE	79	23,080	1,236	4.4	3.2-5.7	97.3	<0.001
KK	118	26,323	408	3.5	2.8-4.1	87.2	<0.001
MBA	1	678	7	2.5	0.5-4.6	…	…
Not mentioned	20	3,080	78	4.5	2.2-6.8	83.8	<0.001
PCR	5	5,417	241	6.9	2.7-11.2	97.3	p = 0.001
Ritchie	1	570	9	1.6	0.5-2.7	…	…
Roman	1	311	10	3.2	1.1-5.3	…	…
SAAF	1	167	7	4.2	0.8-7.6	…	…
Brine concentration + DA	1	1,059	7	0.7	0.1-1.3	….	…
Baermann + KAP	7	387	82	18.8	7.9-29.7	86.5	<0.001
Baermann + KK	1	504	65	12.9	9.9	15.9	…
Baermann + Ritchie	1	1,526	101	6.6	5.3-7.9	…	…
Bailenger + DA	1	240	5	2.1	0.0-4.1	…	…
DA + Formol saline	2	150	8	5.3	1.2-9.4	0	p = 1.000
DA + MFC	1	300	99	33.0	27.5-38.5	…	…
DA + PCR	1	98	3	3.1	-1.0-7.1	…	…
DA + Ritchie	2	2,726	24	5.9	-5.8-17.5	94.3	<0.001
DA + Willis	1	392	4	1.0	-0.1-2.2	…	…
DA+FE	22	20,692	404	2.8	2.0-3.7	88.5	<0.001
FE + KK	3	4,866	94	1.9	0.8-3.1	88.5	<0.001
FE + Ritchie	1	161	7	4.4	0.9-7.8	…	…
KK+MIFC	1	249	83	33.3	27.3-39.4	…	…
KK + Ritchie	1	1,001	14	1.4	0.6-2.2	…	…
Baermann + Coproculture + DA	10	411	11	3.7	0.8-6.7	0	p = 0.855
Baermann + DA + KK	1	782	12	1.5	0.6-2.5	…	…
Baermann+KAP+PCR	1	256	56	21.9	16.6-27.1	…	….
DA + Graham + KK	1	360	1	0.3	-0.5-1.1	…	…
DA + FE + FM	1	4,836	400	8.3	7.5-9.1	…	…
DA + KK + Ritchie	3	1,724	22	1.2	0.6-1.8	0	p <0.001
DA + Lugol + Kato-Muira	1	343	42	12.2	8.6-15.9	…	…
DA + Ritchie + Willis	3	210	1	1.4	-2.4-5.1	…	…
DA+FE+KK	1	551	19	3.5	1.8-5.1	…	…
Baermann + DA + Graham + KK	6	2,220	44	2.2	1.4-3.0	14.5	p = 0.322
KAP	1	112	38	33.9	24.8-43.1	…	…
Baermann+FLOTAC+FE+KK+KAP	1	292	37	12.7	8.7-16.7	…	…

BC: brine concentration, DA: direct analysis, FE: formol-ether, KK: Kato-Katz, KAP: Koga agar plate, MBA: Multiplex bead assay, MFC: merthiolate-formaldehyde concentration, MIFC: merthiolate iodine formaldehyde concentration, SAAF: sodium acetic acid formalin
